# Effect of high hydrostatic pressure on seeds *Carica papaya*

**DOI:** 10.1186/1753-6561-8-S4-P131

**Published:** 2014-10-01

**Authors:** Alan Costa Sarcinelli Santos, Antônio Alberto Ribeiro Fernandes, Patrícia Machado Ribeiro Fernandes

**Affiliations:** 1 Núcleo de Biotecnologia, Universidade Federal do Espírito Santo, Vitória, Espírito Santo, Brazil

## 

The breaking of seed dormancy induced by stress [1] is extremely important with regard to the acceleration of germination process [1,2]. Abiotic stress processes, just like high hydrostatic pressure (HHP), are applied in several biotechnological studies, mainly because HHP induces changes in physiological and biochemical processes [3]. Pretreatment with a stress induction tool can be lethal or induce the synthesis of protective factors. When the stress does not kill the organism, it is called sublethal stress, and it can even increase the resistance to other stresses. High hydrostatic pressure is an important model in stress response study, for it has the ability to change the plasma membrane fluidity so as to keep the organism alive, which allows studies on the metabolic changes. Water is used as the medium of fluid pressure transmission since it has lower compressibility and greater compatibility, resulting in less risk of contamination. In our experiment, *Carica Papaya *seeds were put into a polyethylene tube containing water, the system is then closed in a capsule of high resistance steel, and suffers pressures between 10 and 400 MPa. The seeds were set to germinate *in vitro*, while the pressure transfer fluid present in the capsule was used to analyze abscisic acid per HPLC. The HHP generated positive effects due the seed hydration, which reflected in the percentage and speed of germination [4]. The begin of the seeds germination subjected to HHP was anticipated to 70 hours due to greater water retention, characterized by the increasing of weight (about 2.8 times greater when compared to dry weight), followed by germination values between 70-90%, while the seeds subjected to ambient pressure (control group) showed hydration of about 2.4 times and germination around 55-65%. The ambient pressure (0.1 MPa) showed lower water retention, resulting in lower mean percentage and germination speed when compared to HHP treatments. The abscisic acid (ABA) is closely related to tolerance to stress conditions (HHP) and its relation to gibberellin (GA) is crucial in the process of germination. The pressure transfer fluid has proven useful for the detection of abscisic acid, characterized by the presence of chromatograph peak with retention time equals to analyses of samples fortified with industrial ABA. The analyses of the HPLC chromatograms demonstrate changes in the composition of the water used as medium of pressure transfer. The seeds undergoing HHP pressure showed through gas chromatogram, differences between the concentrations of protein, peptides and phenol, when compared to the seeds subjected to hydrostatic control group. The balance between the concentrations of abscisic acid and gibberelin is related to germination. The presence of ABA in fluid of pressure transfer indicates a factor of germination capacity of seeds subjected to HHP stress. HHP stress is an effective biotechnological tool in the search for new mechanisms of stimulation of *Carica Papaya *seeds in order to provide improved germination and breaking of dormancy. Some data obtained in this experiment were omitted because due to patent submission.
